# Occupancy Monitoring Using BLE Beacons: Intelligent Bluetooth Virtual Door System

**DOI:** 10.3390/s25092638

**Published:** 2025-04-22

**Authors:** Nasrettin Koksal, AbdulRahman Ghannoum, William Melek, Patricia Nieva

**Affiliations:** 1 Mechanical and Mechatronics Engineering Department, University of Waterloo, Waterloo, ON N2L 3G1, Canada; wmelek@uwaterloo.ca; 2 Department of Chemical Engineering, Qatar University, Doha P.O. Box 2713, Qatar; aghannoum@qu.edu.qa

**Keywords:** BLE, RSSI, occupancy monitoring, neural network, deep learning, hybrid learning

## Abstract

Occupancy monitoring (OM) and the localization of individuals within indoor environments using wearable devices offer a very promising data communication solution in applications such as home automation, smart office management, outbreak monitoring, and emergency operating plans. OM is challenging when developing solutions that focus on reduced power consumption and cost. Bluetooth low energy (BLE) technology is energy- and cost-efficient compared to other technologies. Integrating BLE Received Signal Strength Indicator (RSSI) signals with machine learning (ML) introduces a new Artificial Intelligence- (AI-) enhanced OM approach. In this paper, we propose an Intelligent Bluetooth Virtual Door (IBVD) OM system for the indoor/outdoor tracking of individuals using the interaction between a BLE device worn by the occupant and two BLE beacons located at the entrance/exit points of a doorway. ML algorithms are used to perform intelligent OM through pattern detection from the BLE RSSI signal(s). This approach differs from other technologies in that it does not require any floorplan information. The developed OM system achieves a range between 96.6% and 97.3% classification accuracy for all tested ML models, where the error translates to a minor delay in the time in which an individual’s location is classified, introducing a highly reliable indoor/outdoor tracking system.

## 1. Introduction

Occupancy monitoring (OM) solutions are becoming integral to developing smart control and Internet of Things (IoT) systems. In this field of study, occupancy behavior (OB), including both occupancy presence/absence and occupancy interaction with an environment, are key [1]. The representation of OB is necessary in the modeling and development of OM methods.

**Figure 1:**
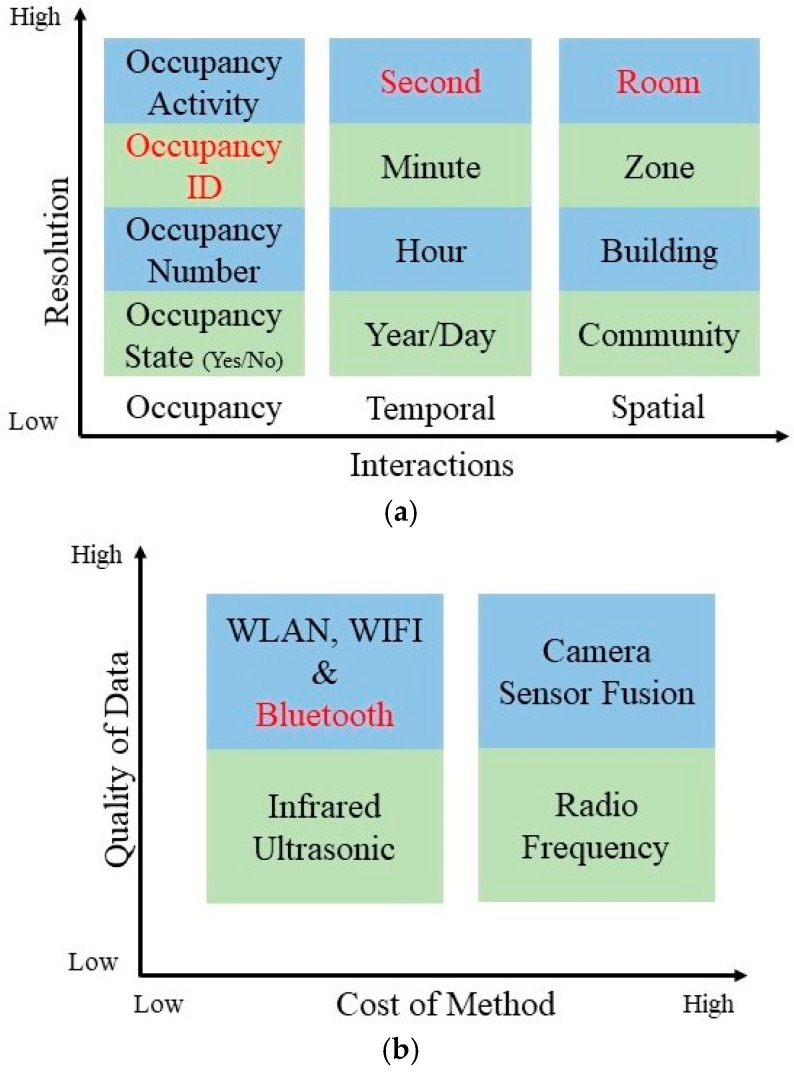
(**a**) Resolution of OM systems, and (**b**) Strength and weakness of sensors that are in use of recent OM systems in literature. The red font indicates the resolution of our IBVD system and/or the sensor technology used in it.

Measuring active occupancy interaction in IoT applications requires a special design and can provide information about the environment, such as behavior and tasks performed by occupants, type of space, date, and time [2,3]. To measure or estimate parameters from an environment with a certain occupancy behavior, the resolution of an OM system is usually grouped into three different types of interactions: (1) occupancy status, (2) temporal information, and (3) spatial information, as presented in Figure 1a. Many hardware (sensing) systems are available in the literature, and they can produce different occupancy resolutions within the taxonomy shown in Figure 1b, which is based on the quality of sensed data as well as the cost of the OM system. Within them, camera-based systems are exceptionally popular in IoT applications due to their ability to provide high-precision information about occupancy activity in every processed camera frame. Depending on the software deployed and the number of units used in camera-based OM systems, not only can occupancy interaction monitoring be realized, but also the exact location of occupants [4,5]. Compared to others, OM systems using cameras offer relatively high data quality in occupancy detections/estimations. However, these systems have some drawbacks, such as higher computational complexity, privacy concerns, high cost, or vision occlusion. PIR and ultrasonic sensors are other electronic sensing devices that are utilized in occupancy estimation models. They provide lower occupancy information and can detect any motion by using infra-red radiation information, which is emitted by the heat energy of objects, and the wavelength of infra-red radiation changes from moving objects [5]. The ultrasonic sensor-based solutions work based on three physical principles: Time of Flight (TOF), Doppler effect, and attenuation of sound waves [6]. However, even though PIR and ultrasonic sensors offer a low-cost and easy-to-deploy solution in some related applications (Figure 1b), they also have drawbacks, such as the possibility of missing occupants who are stationary, narrow sensing cone, low tracking accuracy when the number of occupants in the environment increases, and “False-on” detection errors due to environmental effects such as air turbulence from the HVAC system [5]. Another sensing methodology that is used for occupancy detection and estimation systems is WIFI technology. WIFI-based systems can be an efficient tool to detect the number and ID of occupants in indoor environments with a high degree of data accuracy (Figure 1b). However, WIFI-based solutions have limitations, such as the cost of required infrastructure and the likelihood of generating multiple occupancy detection signals for occupants who carry more than one WIFI-powered device (multiple phones, tablets, etc.) [7,8].

The most used technology to supply OB in state-of-the-art smart control and IoT systems is Bluetooth low energy (BLE). BLE has gained much attention in these applications because of its low power consumption and cost efficiency. When compared to classical Bluetooth technology, which utilizes multi-advertising channels, a major improvement in BLE includes a fast and energy-efficient neighbor discovery process (NDP) design, which uses only three special advertising channels for neighbor discovery [9]. Compared to other sensor technologies, BLE devices have significant advantages in terms of size, cost, energy consumption, ease of deployment, and long operating life, which, for some applications, can span several years [10].

The main sensory readout for BLE devices is the Received Signal Strength Indicator (RSSI). The loss of RSSI signal propagation in space, known as path loss, is utilized in the path-loss distance formula to model the estimated distance between advertising and receiving devices of localization and monitoring systems. However, the RSSI signal depends on the propagation of radio frequency (RF) waves, which are affected by fading, shadowing, and multi-path effects [11]. Chapre et al. studied the factors that influence RSSI fluctuations/variations, which include antenna direction/type, the distance between transmitter and receiver, measurement time/period, other RF operations nearby, human user presence/absence/mobility, and building types/materials [12]. Hence, in mobile applications, the received signal strength (RSS) quality of BLE devices is easily impacted by these factors and will likely become attenuated rapidly as the distance between transmitter and receiver increases.

Algorithms for BLE technology-based indoor positioning systems in the literature mainly use indoor localization and fingerprinting methods [13,14]. These methods are special positioning solutions that track the position information of people or objects within an indoor environment by using a network of sensing devices. They have been sufficiently studied for the development of BLE-based positioning systems with accuracies between 1 and 3 m. Indoor localization methods offer different levels of positioning accuracy, which are directly affected by RF propagation. They consist of two key approaches: (a) range-based and (b) angle-based. The range-based approach uses a path-loss formula to determine the relationship between RSSI and distance, which is used in indoor localization systems for distance calculation or triangulation. This approach presents a challenge in terms of the ability to define a suitable estimation model because of RF propagation [11]. Svecko et al. investigated the effects of antenna design (circle and parallel types) on range-based distance estimation. This study found that different types of antenna placements directly affect the quality of distance estimation due to RF propagation [11]. The angle-based approach can improve localization accuracy by conducting geometric calculations using known beacon nodes and measured angle of arrival (AOA) [15]. Fingerprinting methods provide better positioning accuracy results than indoor localization methods, and their estimation accuracy relies on the number of beacon nodes deployed in application environments. However, to achieve sufficient localization accuracy, a certain number of source nodes, capturing enough signals, and/or an estimation algorithm with a huge amount of RSSI data are needed [16].

Contrary to the reported accuracy levels used in positioning systems in the literature, some IoT applications require more precise position estimation in complex and dynamic environments. Even though increasing the number of beacon nodes can be an alternative solution to improve localization accuracy in BLE-based positioning systems, it can trigger discovery latency in communication and can increase the overall system deployment cost. Due to the drawbacks of recent positioning systems, such as inconsistency, low accuracy, high computational cost, higher system cost, or need for prior environmental knowledge, utilizing OM systems with high resolution can be an attractive and efficient solution to localize human occupants in various IoT applications such as health monitoring [17], HVAC [18] or smart buildings [19].

OM systems are developed to utilize information from the environment that falls into one or more of the following three classes: analytical, data-driven, and knowledge-based [20]. Analytical methods in OM focus on the physical interaction between occupants and the environments. These methods directly utilize information that relates sensed variables from the environment to occupants’ presence/absence [20]. Data-driven methods for OM systems use ML models that are designed to reveal hidden patterns in sensed data with the purpose of providing occupancy information without the need for an analytical model [20]. Several data-driven techniques have been developed and used to detect occupants in indoor environments. The data-driven techniques use ML tools such as Artificial Neural Networks (ANNs) [21], Convolutional Neural Network (CNN) [22], Long Short-term Memory (LSTM) [23], Support Vector Machines (SVMs) [24], and Hidden Markov Models (HMMs) [25]. Knowledge-based approaches utilize specialized information encoded in production IF-THEN rules to develop OM solutions. Some knowledge-based approaches introduced in the literature include rule, thresholding, and occupancy/environmental knowledge-based [26].

Even though some solutions for OM problems in the literature are intelligent [1,10,11,12,13], they do not combine BLE technology and ML methods, and/or they often require knowledge of the environment in which the system is deployed [27]. The use of ML models in OM problems can provide more robust accuracy without having to deal with the effects of hardware-related drawbacks. This is because ML models have the ability to model complex signal feature relationships between the environment and the sensing device. Unlike data-driven ML approaches, analytical and knowledge approaches can perform poorly under the aforementioned drawbacks without additional system maintenance or model optimization. Therefore, specific ML models can be utilized to improve the performance of BLE-based OM systems by eliminating the effects of RSSI fluctuation that exist due to RF propagation in BLE technology. Furthermore, because of the ability to extract patterns in preceding data, ML-based models offer the versatility that allows the generalization of OM solutions, which can be easily adapted to new and previously unseen data.

In this paper, an Intelligent Bluetooth Virtual Door (IBVD) system that provides an intelligent OM solution with high-quality OB data using low-cost BLE technology and ML methods is proposed. The IBVD system utilizes long short-term memory (LSTM), Gated recurrent unit (GRU), and hybrid (LSTM+GRU) ML models, all special versions of recurrent neural networks (RNNs). Performance evaluations through comparative studies are conducted for all these models to show the effectiveness of each ML method when integrated into the proposed IBVD system, which is validated for a pilot office application. In terms of occupancy, temporal, and spatial information metrics, the proposed system offers high OM resolution with unique ID tracking, which is utilized in the proposed machine learning (ML) models instead of low OM resolution data such as event counting. The novel and comprehensive OM approach presented here can be deployed to detect the flow of human movement through strategic pathways, such as entrance and exit points of rooms, hallways, and stairs, without any prior knowledge of the indoor environment and/or floor plan and with fewer BLE beacon nodes. It also provides high OM resolution through unique and anonymous device ID tracking that can be easily used for one or multiple occupant cases. Multi-occupant algorithms can be advantageous in complex applications such as health monitoring and smart home/office management.

## 2. Problem Statement

**Figure 2:**
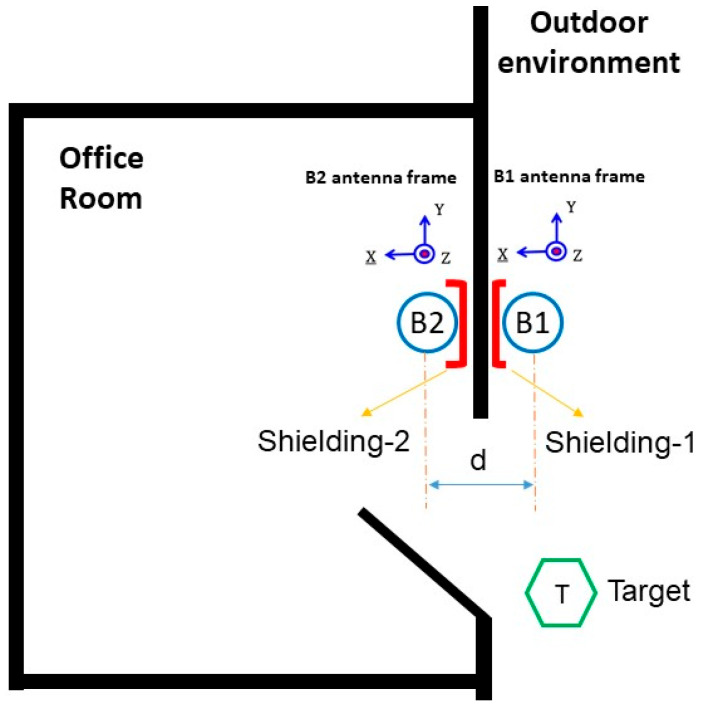
Two BLE beacons based on IBVD setup in the floorplan of our pilot office room.

The IBVD system for the pilot office room studied in this paper is shown schematically in Figure 2. It was developed using two BLE beacons, B1 and B2, positioned at the entrance and exit of a room to form a “virtual door” without requiring precise positioning hardware. The BLE beacons interact with occupants wearing BLE-enabled wearable devices, referred to as targets T, which broadcast anonymous IDs. The system operates by monitoring the Bluetooth signals emitted by the T devices during movement near and through the door path.

The two BLE beacons, B1 and B2, are mounted on opposite sides of the doorway, positioned as closely as possible, with both antennas facing the same direction. Faraday shielding is applied to the wall behind each beacon to minimize interference from external signals and to shape the signal profile within the sensing area. The beacon located inside the room is partially enclosed in a cup-shaped shield, which provides circumferential shielding. The beacon outside the room has shielding material mounted behind it, flat against the wall. This arrangement enhances the contrast in RSSI signal behavior received during transitions without introducing abrupt or artificial signal fluctuations. The only features fed into the machine learning models are the raw RSSI values received by B1 and B2 from the wearable device. As an individual approaches the doorway, the RSSI readings for both beacons increase. When the person enters the room, B2 (inside) continues to receive a stronger signal, while B1 (outside) begins to show a drop. During exit, this pattern is reversed: the RSSI at B2 decreases while B1 increases, followed by a simultaneous decline as the person moves farther from the doorway. These time-series patterns allow the models to learn and infer between indoor and outdoor positions. Because of the simple two-feature input, the system relies entirely on the temporal dynamics and relative differences in RSSI rather than complex feature engineering to make occupancy monitoring (OM) decisions. A sufficient number of scenarios were collected to create labeled training and test datasets, which were then used to design, validate, and compare the machine learning models.

## 3. Methodology

A novel solution is presented to tackle the problem described in the previous section. The aim is to provide occupancy activity monitoring by using the virtual door setup and the BLE-enabled target device in the designated pilot office room shown in Figure 2. The target device activity is observed for multiple indoor-outdoor scenarios, and data are gathered to capture the room occupant’s daily activities during a pre-determined OM period. Training and test data sets needed are then generated to develop multiple ML learning models and compare their levels of accuracy in occupancy activity classification.

### 3.1. Virtual Door Setup

**Figure 3:**
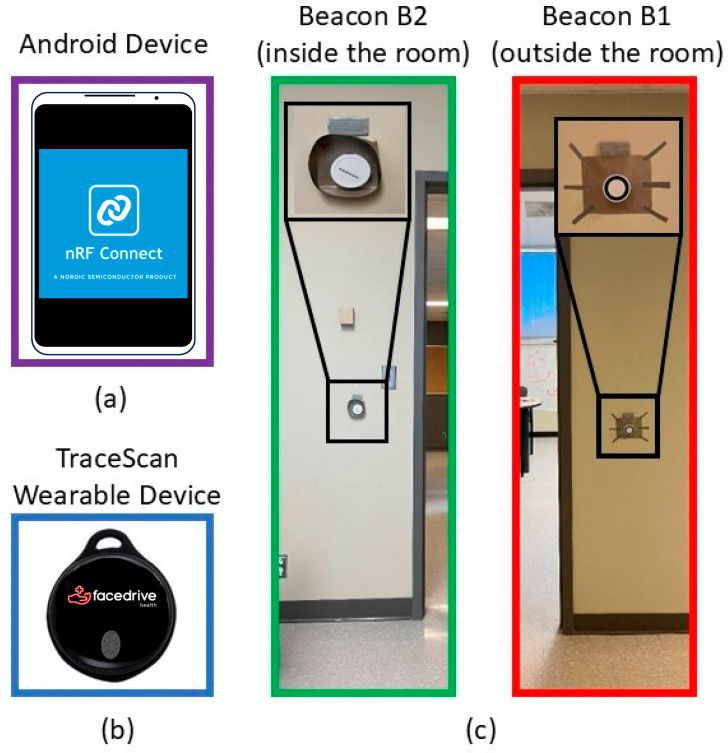
Data collecting and IBVD setup components: (**a**) Android device used to reach 2 beacons for reading and collecting RSSI data, (**b**) Target Wearable Device, TraceScan from Steer Technologies, Inc., (**c**) 2 beacons (inside/outside the room) of IBVD system.

As shown in Figure 3, the virtual door setup used to create the desirable RSSI pattern uses two K8 waterproof BLE beacons manufactured by KKM Smart Solutions, China. The K8 beacon is designed for indoor localization solutions, commercial advertising, and proximity marketing. It has BLE 5.0 technology with an ultra-low-power chipset, the nRF52 series, a waterproof long battery life, and a 2.4 GHz radio signal broadcasting at regular and adjustable intervals.

The targeted wearable device (TraceSCAN) used in this project has been customized by Steer Technology Inc., Canada. The device is based on BLE technology and is a low-energy wireless wearable that can be deployed as a component of Internet-of-Things-enabled platforms. The TraceSCAN device used here comes in the form of necklace tags, which are called wearables. At the heart of the wearable hardware, there is an nRF52 System on Chip (SoC) from NORDIC Semiconductors. The nRF52 series is equipped with generation 5.0 of BLE Radio Front Ends (RFE) and operates in the Industrial, Scientific, and Medical (ISM) spectrum band of 2.4 GHz. The device can provide data at the following rates: 1 Mbps and 2 Mbps, with configurable Tx Power: −20 to +4 dBm (configurable in 4 dB steps), and sensitivity: −96 dBm @1 Mbps and −89 dBm @2 Mbps. The TraceSCAN device also has a smart design platform to enable device configuration for multiple IoT applications ranging from a simple BLE-enabled device finder to a real-time locating tag.

To attenuate the Bluetooth RSSI signals created by B1 (inside) and B2 (outside) forming the virtual setup (see Figure 2), the backsides of both K8 beacons are enclosed by Faraday shielding material made of TitanRF Faraday Fabric developed by MOS Equipment, Inc., USA. The TitanRF Faraday Fabric uses a copper and nickel composition to achieve an average attenuation of 80–120 dB from low MHz signal frequency all the way to 40 GHz. Hence, a clear divergence in RSSI magnitude occurs whenever an occupant wearing the targeted wearable (T) enters and/or exits the room.

### 3.2. Data Collection and Scenarios

The data set generation for the ML algorithms training and testing steps includes data pre-processing and labeling. Since the K8 beacons have a nRF52 chipset and need compatible software, nRF Connect for Mobile (Version 4.26.1) from an Android phone (see Figure 3) is utilized to reach the beacons and read the RSSI data. The nRF Connect for Mobile was built by the Norwegian company Nordic Semiconductor. The algorithm used for data collection using nRF Connect is described below (Algorithm 1):
**Algorithm 1** Algorithm for Data collection using nRF ConnectAt each time instant *t* > 0**Set:** Activity start inside or outside of pilot office room**Collect:** RSSI of BLE Beacon-1/Beacon-2 received from target BLE wearable device**If** the target device changes its environment**Switch** activity inside to outside the room or activity outside to inside the room, and RSSI data collection**End**

This algorithm is executed to collect each one of the datasets needed to describe different occupant movement scenarios created using the virtual room environment shown in Figure 2. These scenarios capture many realistic human-room entering and/or exiting behaviors. During a collection process, each scenario’s start/end times and environment change time stamps are labeled by this algorithm, which utilizes the Android phone with nRF Connect for Mobile.

To obtain sufficient RSSI datasets for training and testing the ML models, 48 different human-room activity scenarios were created with 5 repeated trials in each for a total of 240 data sets. These 48 scenarios were created based on the following human-room interaction cases: entering/exiting, walking long/short distances from outdoors to indoors, opening/closing doors, short-time stay indoors/outdoors, and standing inside/outside in front of the entrance door while the occupant is wearing the target device as a right or left wristband.

### 3.3. Data Pre-Processing and Labeling

For all collected datasets, RSSI measurements from the beacons encounter sudden fluctuations and excessive noise. This is due to the fact that all devices (two beacons and the target device) have BLE-type technology. In addition, the target device is usually mobile, which creates motion artifacts in the measured RSSI signals. It is expected that environmental factors such as human movements, electromagnetic interference from other nearby equipment and devices, and receiver antenna orientation [28] can also cause fluctuations and noise in RSSI measurements.

To reduce the noise and smoothen the RSSI signals of all datasets, one of the most commonly used filtering methods in digital signal processing, the moving average filter, is selected to smoothen the collected RSSI data. This filter works by averaging the number of points from the input signal to produce each point in the output signal. The Moving average filter output is computed as

(1) $$y\left(i\right)=\frac{1}{M}\sum_{j=0}^{M}x\left(i+j\right),$$

where *x*, *y*, and *M* are the input signal, the output signal, and the number of averaging points, respectively.

After pre-processing all collected datasets, a reliable labeling process to generate the classifier output for each feature vector (i.e., RSSI measurement) in each dataset was used. Each feature vector was labeled by one of two classes, indoor/outdoor, as a true output that describes the actual location of the wearable (T) for each scenario described by this feature vector.

### 3.4. Machine Learning Models

**Figure 4:**
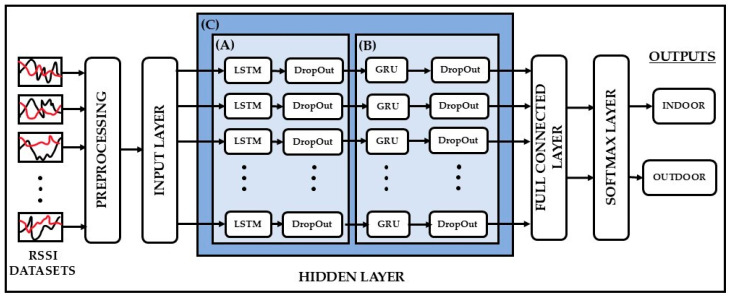
Architectures of LSTM, GRU and hybrid learning models for the classification problem of the IBVD system with the use of three different hidden layers: (**A**) LSTM, (**B**) GRU and (**C**) hybrid.

A recurrent neural network (RNN) is a class of artificial neural networks where the outputs depend on both current inputs and historical data. RNN architectures are mostly used for sequential correlation cases of data. In this paper, three types of RNN occupancy detection models are trained and compared using the RSSI data sets and their occupancy labels (indoor/outdoor) obtained using the data collection algorithm using nRF connect described before. The three architectures of the proposed RNNs are shown in (Figure 4A) LSTM, (Figure 4B) GRU, and (Figure 4C) hybrid (LSTM+GRU) networks. Each one of these models is described next.

#### 3.4.1. LSTM Network Model

LSTM is a special neural network used to solve the long-term data dependency problem in classical RNNs. The advantage of using an LSTM over a classical RNN is its ability to avoid gradient vanishing and explosion by using a hidden memory unit with three gate structures. The ability to store information for future use makes them attractive candidates for developing the indoor/outdoor classifier of the OM system.

A standard memory cell structure of an LSTM network can be described using the following mathematical equations,

(2) $$f_{t}=\sigma (W_{f}[h_{t-1},X_{t}]+b_{f}),$$

(3) $$i_{t}=\sigma \left(W_{i}\left[h_{t-1},X_{t}\right]+b_{i}\right),$$

(4) $$o_{t}=\sigma \left(W_{o}\left[h_{t-1},X_{t}\right]+b_{o}\right),$$

(5) $${\hat{c}}_{t}=tanh\left(W_{c}\left[h_{t-1},X_{t}\right]+b_{c}\right),$$

(6) $$c_{t}=f_{t}⊗c_{t-1}+i_{t}⊗{\hat{c}}_{t},$$

(7) $$h_{t}=o_{t}⊗{tanh(c}_{t}),$$

where $f_{t}$, $i_{t}$ and $o_{t}$ represent three gates: the forget gate, the input gate, and the output gate, respectively. These gates update the following cell states: ${\hat{c}}_{t}$ the candidate’s cell state, $c_{t}$ the current cell state, and $c_{t-1}$ the previous cell state. Also, $X_{t}$, $h_{t}$ and $h_{t-1}$ are the input of current cell, the output of current cell, and the output of previous cell. Therefore, in the LSTM cell structure, the forget gate is responsible for deciding the information forgotten from the previous cell state based on the output of the previous cell and the input of the current cell. The input gate and the output gate provide data for entering and leaving the current LSTM cell. The input gate determines which input values are used to store in the candidate cell state. The output gate determines the value of the current cell state. All three gates have a sigmoid layer with sigmoid activation function σ to decide the output of each gate between 0 and 1, where 1 represents completely keeping the data, and 0 represents completely disposing of the data. $W_{f}$, $W_{i}$, $W_{o}$, $W_{c}$, denote the weight parameters of the forget gate, the input gate, the output gate, and the candidate cell state. $b_{f}$, $b_{i}$, $b_{o},b_{c}$ represent the bias parameters of the forget gate, the input gate, the output gate, and the candidate cell state, respectively.

As illustrated in Figure 4, the LSTM network architecture used in this work consists of an input layer with the pre-processed RSSI variables followed by a hidden layer that contains an LSTM layer and a dropout layer with 200 hidden memory nodes as represented in Figure 4A. In the LSTM network, the dropout layer is applied to avoid overfitting in the modeling process. Then, a fully connected layer is used to connect all hidden memory nodes to a desired output size as a regression layer. As a last layer of the LSTM network, a SoftMax layer is utilized to apppoint decimal probabilities to one of the indoor/outdoor classes for the prediction.

#### 3.4.2. GRU Network Model

The GRU network model is one of the most widely used RNN models. It has a gating mechanism to learn long-term data dependencies. As in the LSTM model, GRU has also been developed to address the vanishing gradient problem with a simpler structure. However, the GRU uses two gates instead of three. Since a GRU cell structure needs fewer parameters and is simpler, this type of RNN model can offer several advantages over LSTM, such as faster training, less computational power, and, in some cases, the use of less data for model training. The GRU model uses the input gate and forget gate simultaneously in the update gate and the reset gate. Hence, the reset gate and update gates share the responsibility of memory retention/erasing in the GRU cell structure.

A standard cell of a GRU network performs its operation based on the following mathematical equations,

(8) $$z_{t}=\sigma \left(W_{z}X_{t}+U_{z}h_{t-1}+b_{z}\right),$$

(9) $$r_{t}=\sigma \left(W_{r}X_{t}+U_{r}h_{t-1}+b_{r}\right),$$

(10) $${\hat{h}}_{t}=tanh\left(W_{c}X_{t}+U_{h}\left(r_{t}⊗h_{t-1}\right)+b_{c}\right),$$

(11) $$h_{t}=z_{t}⊗{\hat{h}}_{t}+{(1-z}_{t})⊗h_{t-1},$$

where the three gates of the LSTM cell are replaced by two gates with $z_{t}$ being the reset gate and $r_{t}$ being the update gate. These gates modify the following states: ${\hat{h}}_{t}$ the candidate cell state and $h_{t-1}$ the previous cell state. $X_{t}$ and $h_{t}$ are the input and the output of the current cell. Hence, in the GRU cell structure, the reset gate controls the information forgetting based on previous information and the input of the current cell. The update gate determines the information from the previous cell state that will transfer to the candidate cell state. Then, two gates produce the output of the current cell. Two gates have a sigmoid layer with sigmoid activation function σ for compressing the outputs of each gate between 0 and 1. $W_{z}$, $W_{r}$, $W_{c}$ represent the first weight parameters of the reset gate, the update gate, and the candidate cell state. $U_{z}$, $U_{r}$, $U_{c}$ denote the second weight parameters of the reset gate, the update gate, and the candidate cell state. $b_{z},b_{r},b_{c}$ represent the bias parameters of the reset gate, the update gate, and the candidate cell state, respectively.

As stated in the previous subsection and presented in Figure 4, the GRU network architecture is structured with the same layers as used in the LSTM network, extending over the indoor/outdoor prediction. The only difference is a new hidden layer utilized with a GRU layer and a dropout layer with 200 hidden memory nodes, as denoted in Figure 4B.

#### 3.4.3. Hybrid (LSTM+GRU) Network Model

In this work, the integration of LSTM and GRU networks is also studied using a hybrid network model. For this purpose, the objective is to capture the data learning capabilities of both the LSTM and the GRU models in a single framework. Hence, the proposed hybrid model combines the benefits of both LSTM and GRU architectures, leveraging their respective strengths to develop a unified model that potentially improves classification performance over either individual RNN models. 

In the hybrid network architecture, the same layer structure is preserved as in the LSTM and the GRU networks explained in the previous subsections and presented in Figure 4. On the other hand, a new hidden layer is designed and used as represented in Figure 4C. The hidden layer is formed by an LSTM layer, a dropout layer, a GRU layer, and a dropout layer, respectively, and each of them has 200 hidden memory nodes. The operation of this hidden layer is as follows. The LSTM layer, with its dropout layer, produces the first-level prediction outputs. Afterward, the outputs of the LSTM prediction are fed to the GRU layer with its dropout layer to generate the final level prediction outputs before reaching the fully connected layer, and then the SoftMax layer to compute the indoor/outdoor prediction.

### 3.5. Model Evaluation Metrics

Model evaluation is one of the main steps in the development of ML-based models and aims to analyze the model’s predictive performance and its generalization ability. For this purpose, confusion matrix analysis and k-fold-based model evaluation methods are adopted to evaluate the predictive performances for all the proposed RNN-based ML models shown in Figure 4.

#### 3.5.1. Confusion Matrices

Confusion matrices are commonly used to evaluate predictive errors in classification problems. As a first evaluation study, to evaluate the accuracy of classification of all three ML learning models utilized in the IBVD system, the following metrics of the confusion matrices are computed: accuracy, precision, recall, and F1 score.

To compute all the metrics needed to define the confusion matrix for each ML evaluation step, the following terms are defined to describe the classifier output from each RNN when presented with an RSSI input feature vector for a specific device (T) in the IBVD environment: True Positive (*TP*): the predicted class is positive, and the actual class is correct, False Positive (*FP*): the predicted class is positive, and the actual class is incorrect, False Negative (*FN*): the predicted class is negative, and the actual class is incorrect, and True Negative (*TN*): the predicted class is negative, and the actual class is correct. Then, all learning models can be evaluated as following evaluation metrics.

*Accuracy* is the portion of correctly classified data and is defined as

(12) $$accuracy=\frac{TP+TN}{TP+TN+FP+FN}.$$

*Precision* shows the ability to classify labels accurately predicted for each class and is defined as

(13) $$precision=\frac{TP}{TP+FP}.$$

*Recall* is the average of true labels correctly predicted for each class. It is defined as

(14) $$recall=\frac{TP}{TP+FN}.$$

*F*1 is a harmonic mean of *precision* and *recall* in a measure used to check model classification accuracy, and it is defined as

(15) $$F1=\frac{2\times precision\times recall}{precision+recall}.$$

#### 3.5.2. k-Fold Cross Validation

The k-fold cross-validation method aims to evaluate ML models by use of data not seen during model training via the resampling method. Here, in modeling processes, k represents the number of folds into which the whole data needs to be split. In each of the k-fold validation sessions, trained models will be evaluated on how well they perform for data used in their training, as well as how precise each of them is in terms of predicting the correct output label for unseen data or limited data. Even though the use of the whole dataset in training gives the best performances in modeling processes, k-fold helps us design more generalized learning models. Hence, the k-fold cross-validation method can improve the generalization ability of ML models for unseen data. This validation method is also effective in reducing the likelihood of over-fitting the data, which can result in poor model generalization, i.e., higher than desirable model output variance.

**Figure 5:**
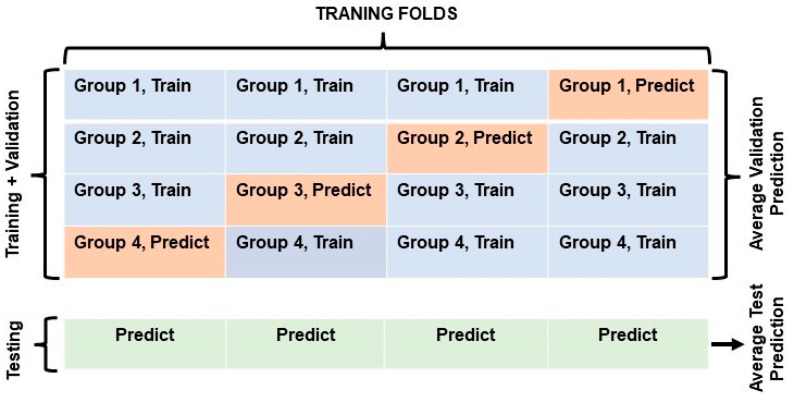
Four-fold data splits used in ML learning modeling. Three groups of all 4-folds represent the training data (60% of all data sets) as colored in blue, and 1 group of 4-folds represents the prediction (validation) data (20% of all data sets) in training as colored in red during 4-fold validation sessions. Each testing data (20% of all data sets) of the sessions is also colored green.

In this study, 80% of all collected data is split into 4-fold, with 3-folds used for training and 1-fold for validation of the RNN model performance. For each of the 4 different training sessions, these folds are alternated, as shown in Figure 5. For testing purposes, 20% of all collected data, not seen by the RNN in each specific training fold, is used to evaluate the generalization performance of each model as also shown in Figure 5.

## 4. Results and Discussion

In this section, the output results of the three different RNN-based ML models, namely LSTM, GRU, and hybrid, applied to the IBVD system for predicting occupancy indoor/outdoor activities in the proposed pilot room are presented, compared, and discussed. All training processes are implemented on a computer equipped with a GeForce RTX 3080 GPU. MATLAB (Version R2022a) was used to build, train, and test all the presented models.

To provide a better performance evaluation, each learning model is designed using the same architectural structure illustrated in Figure 4. Each model has the following hyperparameters: Adam optimizer, a learning rate of 0.001, a batch size of 25, a validation frequency of five, and a maximum epoch size of 200, while training and testing by using the same dataset protocol presented in Table 1. In the RNN architectures, a dropout layer with a similar layer size is also utilized after each RNN layer to reduce overfitting and enhance the generalization ability of models.

**Figure 6:**
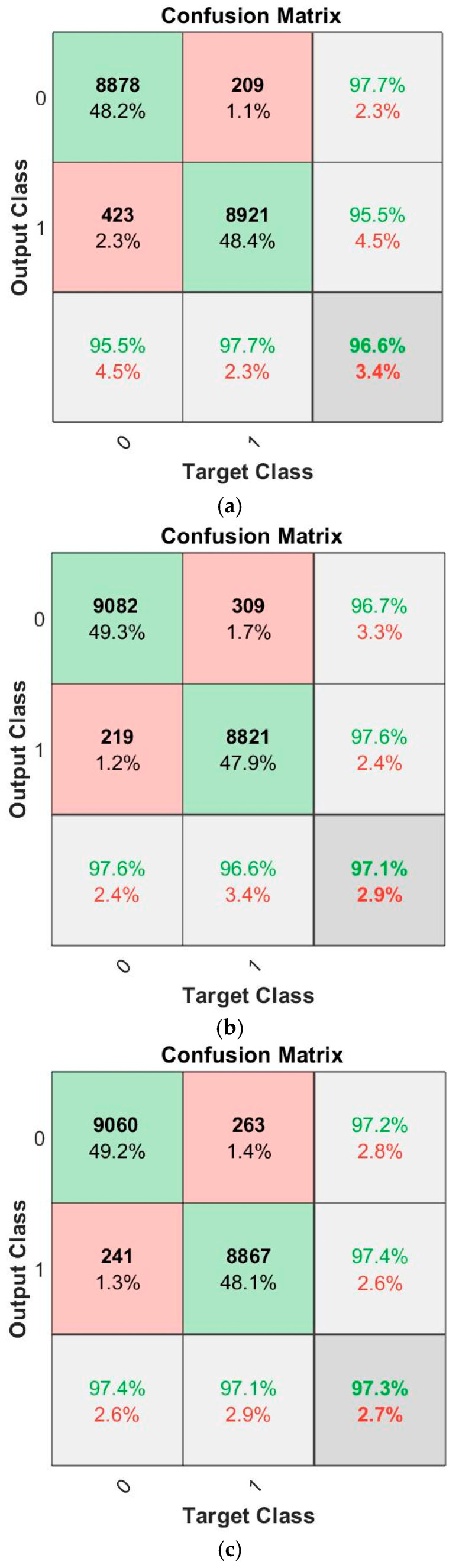
Confusion Matrices of (**a**) GRU, (**b**) LSTM and (**c**) hybrid Models. The 0 and 1 labels represent Indoor and Outdoor classes, respectively. TP, FP, TN, and FN evaluation parameters are obtained from the confusion matrices as follows. TP is represented in green by the intersection of the prediction (output) class 0 and the actual (target) class 0. FP is denoted in red by the intersection of the prediction class 0 and the actual class 1. TN is represented in green by the intersection of the prediction class 1 and the actual class 1. FN is denoted in red by the intersection of the prediction class 1 and the actual class 0. All evaluation matrices are derived by using these parameters. The overall accuracy of each model is represented in dark gray, while light gray is used to represent the precision and recall metrics.

In the confusion matrix-based evaluation, overall accuracies and F1 scores are considered the best evaluation metrics to study the classification accuracy of each of the three ML models utilized. It can be seen in Figure 6 and Table 2 that all three learning models (trained using the datasets for 48 different scenarios, with five trials for each) have achieved exceptional indoor and outdoor classification performances with over 96% accuracy in tracking a single occupant in the pilot room. As expected, overall accuracies and F1 scores show that the hybrid model gives a slightly better classification performance by comparison to the GRU and LSTM models, as seen in Table 2. Since the theoretical structure of LSTM networks allows for recalling long-term dependencies, LSTM and hybrid models both show noticeable improvement in prediction performances by comparison to the GRU model when larger datasets are used in training. The hybrid model also provides better generalization performance based on its overall precision, recall, and F1 scores, as seen in Table 2.

**Figure 7:**
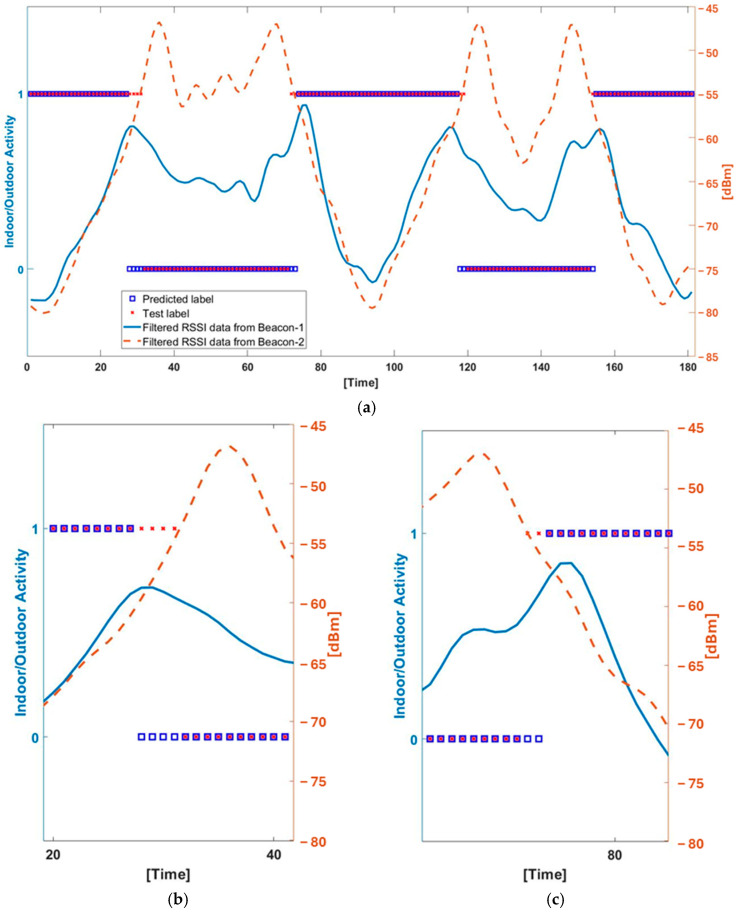
An example Indoor [0] and Outdoor [1] classification performances of LSTM regarding RSSI data [dBm] from both beacon 1 and 2 in the IBVD system (**a**) and zoomed-in views of the first Outdoor to Indoor (**b**) to Indoor to Outdoor (**c**) transitions with classification and RSSI data. This is one of all 240 custom-created scenarios that are used in training/testing protocol. This scenario is a closed door, entering from outdoor and walking from long distance case, and entering/exiting procedure is repeated two-times (0–90 s and 90–180 s) in this scenario.

**Figure 8:**
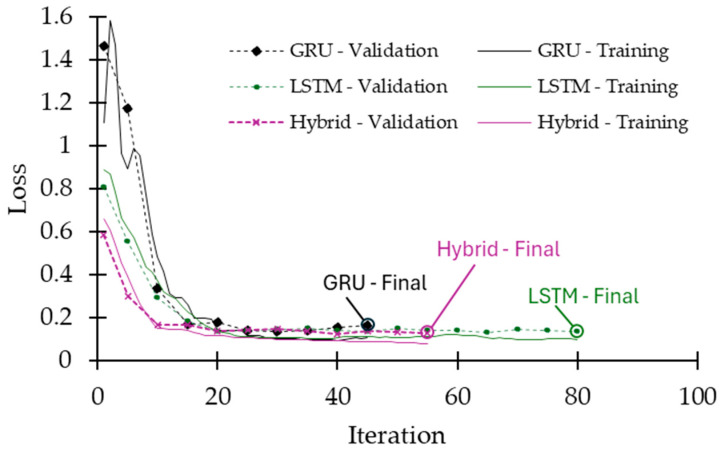
Training and validation loss curves for the GRU, LSTM, and hybrid models. All models exhibit convergence. The hybrid model achieves the lowest final validation loss, indicating better generalization and training stability. The LSTM model follows closely, while the GRU model converges at a higher loss value with more variability in early training.

In addition to the confusion matrix, occupancy monitoring delay (OMD) is evaluated as another metric. OMD occurs at transition regions (indoor-to-outdoor and outdoor-to-indoor) as illustrated in Figure 7. OMD is evaluated since all misclassifications observed from the confusion matrices in Figure 6 occur at critical transition times, and a delay in the prediction of occupancy levels (indoor and outdoor) can be crucial in a real-time application. When considering the total number of misclassifications from the confusion matrices in Figure 6 and critical transitions for 48 different scenarios, the OMD performances of three learning models are 1.65 s for GRU, 1.37 s for LSTM, and 1.3 s for the hybrid models. In other words, LSTM and the hybrid models outperform GRU for the OMD metric. The performance of an ML model on the OMD metric depends on the RSSI signal’s data frequency. Hence, it is anticipated that increasing the frequency of the RSSI-measured signal will enhance classification delay in critical transition times as well as for the overall model(s) classification performance. Figure 8 presents the training and validation loss curves for the GRU, LSTM, and hybrid models. All three models demonstrate stable convergence. The hybrid model shows the lowest final validation loss and the most stable behavior, indicating superior learning efficiency and generalization. The LSTM model also achieves good performance with a slightly higher final loss, while the GRU model converges more slowly and stabilizes at a higher loss value. These trends support the hybrid model’s superior classification performance. However, this comes at the cost of increased computational complexity and resource consumption during training. To prevent overfitting, early stopping was applied using five validation sets for each model. This technique monitored validation performance and halted training when no significant improvement was observed, helping ensure generalization and training efficiency.

In the next evaluation method, k-fold cross-validation is evaluated for all three learning models, which have similar architectural structures and hyperparameters as mentioned above and shown in Figure 5. This validation method allows the comparison of the learning models in terms of their generalization ability while avoiding overfitting on unseen datasets. Table 3 clearly shows that the hybrid model performs more consistently in terms of better classification accuracy on the validation and test sets for each of the k-folds used in training sessions, as well as the best overall classification performance accuracy. It is also observed that the average model training times are 6 min for GRU, 6.44 min for LSTM, and 12.17 min for the hybrid models. Therefore, GRU models require fewer computational resources than the LSTM and hybrid models.

Although the current evaluation was conducted in a single-room setting, the system is inherently designed to support deployment across multi-room environments. The sensing mechanism relies on Bluetooth signal strength received by fixed beacons placed at individual doorways, with Faraday shielding used to minimize cross-directional interference. This setup allows each doorway to be instrumented independently, enabling the system to function reliably in a variety of spatial configurations. Moreover, experiments were carried out in the presence of active Bluetooth devices such as smartphones and laptops, which supports the system’s robustness under real-world interference conditions. While the machine learning models were trained on data collected from a specific layout, the signal features used for classification are highly localized. As a result, changes in the surrounding environment are not expected to significantly impact model performance. Future studies will explore deployments in different building layouts to further evaluate the system’s generalizability and scalability.

## 5. Conclusions

Occupancy monitoring (OM) techniques supply crucial information, such as occupancy number and status, in smart control and IoT systems to improve their application functionalities. Current solutions have limitations and disadvantages, such as the inability to guarantee the privacy of personal data gathered by the IoT-powered device, lack of accessibility, high computation resources needed for deployment, installation difficulties, and/or high overall system cost. In this paper, we propose an IBVD system that implements an OM method using RSSI pattern recognition-based ML algorithms and a BLE-enabled wearable target device with an anonymous ID. To the best of our knowledge, the proposed approach is the first research investigation focusing on the integration of ML algorithms and BLE technologies. The IBVD system is designed for OM in indoor environments without the need for any prior floor plan or environmental knowledge at the time of deployment. Hence, this system can provide a more generic OM solution that can be deployed in different workspaces or indoor environments. The experimental results of all three RNN-based ML models utilized in the IBVD system show that all learning models provide exceptional classification performance compared with other studies reported in the literature. For our collected datasets during the training processes, all three models showed excellent output classification accuracy ranging from 96.6% to 97.3%. When the results of k-fold cross-validation were examined, the hybrid model performed the best in terms of overall accuracy and in classifying data cases not seen during each of the k-fold validation scenarios (above 97% accuracy). Due to the consistent modeling in k-fold validation sessions, it is also postulated that the hybrid model can provide even better classification performance over the other two learning models if a larger dataset were to be used in training. It is also noted that GRU has the fastest training speed because of its simpler structure and fewer model parameters than the other two ML methods.

This study shows that the integration of ML and BLE technologies has the potential to improve existing OM methods while eliminating certain disadvantages of existing commercial solutions. Compared to the state-of-the-art solutions in OM, the proposed IBVD system provides many benefits, including lower hardware cost, ease of deployment, and ease of integration, with the potential to be implemented in many indoor systems to improve their performance. These systems may include HVAC control units, smart offices, smart employee tracking, and home smart systems, among others. In addition to commercial applications, the proposed system can also be exceptionally effective for public safety purposes, such as student tracking systems in schools and health monitoring systems in hospitals. Our next ongoing study is focused on testing the IBVD system in more complex environments, such as multi-room areas or dynamic settings with high occupancy traffic, to evaluate its performance and generalizability under realistic conditions, including obstacles. It also investigates the interference caused by multiple BLE devices operating simultaneously, including scenarios where IBVD systems are present in neighboring rooms in order to validate the IBVD model’s prediction capabilities in environments with signal disruption. Further studies will include the investigation of the performance of the proposed RNN-based ML models using faulty and noisy data obtained from wearables deployed in indoor environments, such as the one considered in this paper. In another potential future study, methods to reduce occupant state classification time delay will be investigated during critical transition times from indoor-to-outdoor and outdoor-to-indoor cases. Another potential avenue for future work will involve conducting real-world experiments to directly compare the performance of the IBVD system with other technologies, such as cameras and WIFI, to provide a more comprehensive evaluation of its effectiveness in practical settings.

## Figures and Tables

**Table 1:** Data training/testing protocol.

Total Data (~105,000 Datapoints)	Usage
60%	Training
20%	Validation in training
20%	Testing

**Table 2:** Classification results of three learning models based upon Precision, Recall and F1.

Model	Accuracy	Label	Precision	Recall	F1
GRU	96.6	Outdoor	95.5	97.7	96.5
		Indoor	97.7	95.5	96.5
LSTM	97.1	Outdoor	97.6	96.6	97.0
		Indoor	96.7	97.6	97.1
Hybrid	97.3	Outdoor	97.4	97.1	97.2
		Indoor	97.2	97.4	97.3

**Table 3:** Four-fold evaluation performances.

Model	Protocol	K1	K2	K3	K4	Average
GRU	Validation	96.6	97.7	97.7	96.0	97.0
	Test	96.6	97.0	97.1	96.9	96.9
LSTM	Validation	97.2	97.9	97.6	96.9	97.4
	Test	97.1	97.2	97.3	96.8	97.1
Hybrid	Validation	97.6	97.9	97.5	97.3	97.6
	Test	97.3	97.2	97.1	97.1	97.2

## Data Availability

Due to the potential commercial value of the new dataset used in this study, we will not be making it open-source at this time. We recognize the significance of open-source datasets for scientific research; however, to protect business confidentiality and future commercial applications, we must maintain exclusive rights to this data.
